# Neurological update: neuroimaging in dementia

**DOI:** 10.1007/s00415-020-10040-0

**Published:** 2020-07-07

**Authors:** Timothy Rittman

**Affiliations:** Department of Neurosciences, University of Cambridge, Cambridge, UK

**Keywords:** Neuroimaging, MRI, PET, Connectivity, Translation

## Abstract

Neuroimaging for dementia has made remarkable progress in recent years, shedding light on diagnostic subtypes of dementia, predicting prognosis and monitoring pathology. This review covers some updates in the understanding of dementia using structural imaging, positron emission tomography (PET), structural and functional connectivity, and using big data and artificial intelligence. Progress with neuroimaging methods allows neuropathology to be examined in vivo, providing a suite of biomarkers for understanding neurodegeneration and for application in clinical trials. In addition, we highlight quantitative susceptibility imaging as an exciting new technique that may prove to be a sensitive biomarker for a range of neurodegenerative diseases. There are challenges in translating novel imaging techniques to clinical practice, particularly in developing standard methodologies and overcoming regulatory issues. It is likely that clinicians will need to lead the way if these obstacles are to be overcome. Continued efforts applying neuroimaging to understand mechanisms of neurodegeneration and translating them to clinical practice will complete a revolution in neuroimaging.

## Introduction

Brain imaging in dementia is undergoing a revolution that is transforming neuroimaging research from merely describing changes in the brain, to understanding what those changes mean. This revolution has been driven primarily by a need for biomarkers to evaluate potential disease modifying treatments, leading to a better understanding of the association between neuroimaging changes and underlying pathology. The effect has been a suite of neuroimaging methods and analytics that help with:

- identifying diagnostic subtypes
- predicting prognosis
- monitoring pathology in vivo.

The benefits of using neuroimaging in this way may find their way to memory clinics in the near future. Neuroimaging for the clinical diagnosis of dementia has traditionally been used to rule out alternative causes of cognitive impairment. Times are changing, and nearly all the diagnostic criteria for neurodegenerative diseases now include neuroimaging as a supportive criterion, and in some cases, such as Frontotemporal Dementia [1], imaging changes are part of the core criteria. However, these criteria remain vague on the specific sequences or measures required to support a diagnosis, usually specifying ‘atrophy’ in a region of interest. As automation and quantification becomes more prevalent to evaluate neuroimaging, it is likely that future criteria will become more specific on the extent of change that suggests a specific diagnosis and the type of neuroimaging required as evidence.

In this review, we discuss a few of the most significant recent advances in neuroimaging and what they mean for our understanding of dementia and the potential relevance for clinical practice. Due to space constraints we cannot cover all of this field including the use of neurophysiology with MEG [2], the application of arterial spin labelling as a promising disease measure [3, 4], and insightful new approaches using combinations of imaging modalities to investigate disease aetiology [5].

## Structural imaging

Since seminal studies of progressive hippocampal atrophy in Alzheimer’s disease in the 1990s [6], structural MRI has been the workhorse of neuroimaging in dementia. Structural imaging has revealed atrophy decades prior to the onset of symptoms in cohorts of people with Mendelian forms of Alzheimer’s Disease [7] and Frontotemporal Dementia [8].

Changes in brain structure continue to provide insight, particularly on the subtypes of disease. For example, the identification of distinct atrophy patterns associated with different rates of disease progression in Alzheimer’s disease and Frontotemporal Dementia [9]. The SuStaIn model used in this work adds to an understanding of clinical heterogeneity in disease progression by associating differential rates of changes over time with specific patterns of atrophy.

Despite its long history, relying on structural MRI for the early diagnosis of Alzheimer’s disease in the form of mild cognitive impairment (MCI) is not currently recommended. A recent Cochrane review found a high false negative rate of 27% and a false positive rate of 29% [10] based on data from 33 studies. Most of the data came from studies looking solely at the hippocampus although overall the data quality was sparse with insufficient data available to establish whether, for example, total brain volume could better differentiate between MCI and Alzheimer’s disease.

Although this seems a disappointing finding, there may yet be more sensitive analytical tools or mathematical approaches using machine learning that can pick up patterns of structural change that may prove useful as a diagnostic biomarker [11].

An alternative approach to identifying a more sensitive imaging biomarker is to use a more powerful scanner, such as 7 T MRI. The “Tesla” refers to the strength of the magnetic field of an MRI scanner, with 1.5 T the one most often used in clinical practice, and 3 T MRI scanners being used routinely for research at academic centres. Although 7 T scanners are less common, shared protocols are helping to facilitate data-sharing to build larger cohorts between 7 T centres, such as those published by the UK7T network [12]. The stronger magnetic field is particularly useful in identifying vascular abnormalities, including cerebral microbleeds, which was the focus of much of the early work with7T MRI in neurodegeneration [13, 14]. One study found that 78% of people with early Alzheimer’s disease/MCI had cerebral microbleeds [15], presumably relating to some form of amyloid angiopathy. These findings strengthen the argument for an early and important role for vascular abnormalities in Alzheimer’s disease.

This increased imaging power offered by 7 T MRI, also allows for better resolution of small structures in the brain, including subfields of the hippocampus. Analysis of the hippocampus in this way has suggested that the pre-subiculum is the earliest subfield to be involved in Alzheimer’s disease [16], and that atrophy is greatest in the pre-subiculum and subiculum in this condition—a finding that is supported by other imaging approaches as well as pathological studies [17, 18]. This level of resolution is now getting to a point where we may be able to image pathology in vivo, a claim strengthened by the possibility of identifying cortical layers in vivo using 7 T MRI [19].

Other small structures relevant to less common neurodegenerative diseases are now also amenable to measurement using 7 T MRI, for example the locus coeruleus in progressive supranuclear palsy [20]. An atlas of the locus coeruleus in an older population is freely available for this purpose (https://www.nitrc.org/projects/lc_7t_prob/).

High-field MRI is therefore playing an increasing role in evaluating structural brain changes at ever increasing levels of resolution which is now approaching that of delineating pathology in situ.

## Positron emission tomography (PET)

The pathology of dementia in vivo promises to be revealed by the fastest growing player in dementia imaging: Positron Emission Tomography (PET). PET ligands for beta-amyloid are now well established [21], though their use in elderly populations is limited given the high rates of false positives; in an Australian cohort of cognitively normal people the number of positive beta-amyloid scans was 18% at 60–69, rising to 65% over the age of 80 [22]. To determine the clinical value of beta-amyloid PET, a real world study of 11,409 participants in the US aims to assess its utility in memory clinics (https://www.ideas-study.org/). The study is ongoing, but initial results demonstrate that an amyloid PET scan led to a change in patient management in 60.2% of people with MCI and 63.5% of people with dementia, with approximately three quarters of the change being the commencement of a drug for Alzheimer’s disease [23]. It is hard to know whether this is useful in that a potential biomarker has had such a large impact, or rather that there are concerns given the high false positive rate could lead to over-diagnosis and unnecessary treatment. In this respect, the planned follow-up studies to assess whether there is a beneficial clinical outcome to this widespread use of amyloid PET and the associated change in clinical practice is much anticipated.

Ligands targeting proteins other than beta-amyloid have also been developed, with tau being the most advanced. First-generation ligands such as AV-1451 [24] are particularly useful in Alzheimer’s disease which is associated with abnormally hyperphosphorylated and misfolded tau that contains both 3 and 4 repeats of exon 10 of the MAPT gene, so-called 3R/4R tau. Other tauopathies such as progressive supranuclear palsy (4R tau), corticobasal degeneration (4R tau), frontotemporal dementia (3R tau) and chronic traumatic encephalopathy (3R/4R tau) are characterised by different and distinct types and conformations of abnormally folded tau [25, 26]. The tau PET ligands bind less avidly to these alternative isoforms forms of tau [27]. Furthermore, there are issues of off-target binding, for example AV-1451 binds to the TDP-43 protein found in Semantic Dementia, motor neurone disease and a proportion of people with frontotemporal dementia [28, 29], and with monoamine oxidase in the basal ganglia [30, 31]. Second generation tau ligands are emerging with less off-target binding, but none have yet demonstrated good affinity for non-Alzheimer tauopathies [32]. Despite their limitations, the first-generation tau ligands do show changes in expected brain regions in tauopathies including progressive supranuclear palsy [33] and apraxia of speech (a subtype of non-fluent variant primary progressive aphasia) [34]. Therefore, at the present time, tau PET is most relevant for Alzheimer’s disease as a potential diagnostic biomarker but may be useful for tracking changes in pathology for both Alzheimer’s disease and other tauopathies.

Other PET ligands target potentially important disease mechanisms. Inflammation is a very active research topic in dementia at the present time, in particular the role of microglia and their function in driving the disease state [35]. Activated microglia express the protein TSPO that has been a target for PET ligands [36]. Applying the first generation of these ligands has suggested an early role for inflammation in a number of neurodegenerative diseases, including dementia with Lewy bodies [37], Alzheimer’s disease [38, 39], corticobasal syndrome [40], progressive supranuclear palsy [41] and frontotemporal dementia [42]. The second generation ligands are more specific, but limited by the fact that approximately 30% of the population has a genetic variation meaning the ligand will not bind to TSPO [43].

Other ligands have emerged as possibly being useful in dementia states, for example UCB-J to measure synaptic density [44]. The UCB-J ligand has been looked at in Alzheimer’s disease [45] and revealed reduced synaptic density in the hippocampus that was correlated with episodic memory, although this study only used a group of 10 people.

Perhaps surprisingly in this field, there has not yet been a successful ligand to target alpha-synuclein as found in Parkinson’s Disease, Dementia with Lewy Bodies and Multiple System Atrophy, although efforts are ongoing [46, 47].

PET will continue to add to our knowledge of human in vivo neurodegeneration as ligands improve and the range of targeted ligands broadens. The relationship of PET to cognition and neuropathology needs to be clarified further, but one imagines this field will mature very quickly. It is likely to provide a source of biomarkers for trials of disease modifying treatments.

Despite revealing pathology, PET does not necessarily explain why specific brain regions are affected or how the brain compensates for the presence of pathology, something which connectivity analysis studies promises to shed light on.

## Structural and functional connectivity

The field of connectivity uses neuroimaging to examine connections between brain regions, either functional connections [48, 49] by examining time series data (functional MRI, EEG, MEG); or with structural connections (diffusion tensor imaging, cortical thickness). The importance of brain networks was highlighted by pioneering work demonstrating that brain networks are a template for atrophy in various neurodegenerative diseases [50, 51], for example the default mode is associated with Alzheimer’s disease [52] and the salience network with frontotemporal dementia [53].

It has been tempting to link these networks to the theory of protein templating and spread of pathological proteins through connected brain regions in a prion-like fashion [54], and indeed the distribution of tau has been linked to functional networks [55]. This concept of ‘prion-like’ spread posits that abnormally conformed proteins cross synapses between connected brain regions to cause normal proteins to become abnormal in the “infected” region. Alternatively it may be that regions within these brain networks share a common susceptibility to disease linked to neurodevelopmental changes laid down through genetic variance that associates with later disease states [56]. Ultimately, it is likely that both spread and susceptibility play a part in neurodegeneration, but have different roles in different disorders; for example we have used functional connectivity and PET to examine the distribution of tau in neurodegeneration, finding that the pattern in Alzheimer’s disease was more in keeping with trans-synaptic spread, and in PSP was more in keeping with susceptibility of metabolically active regions [57].

As well as providing susceptibility to disease, there is some evidence that brain networks can be helpful in compensating for the effects of early pathology, maintaining cognition in the long presymptomatic phase during which atrophy is detectable on scanning but cognition remains normal [58]. This is in line with the idea of cognitive reserve, that early life education, social interactions and genetic factors protect the brain from the effects of dementia [59, 60]. Cognitive reserve appears to be associated with a specific network of brain regions, though the implications for neurodegeneration are not yet fully understood [61]. However, FDG-PET studies have demonstrated a role for cognitive reserve in Alzheimer’s disease [62], Dementia with Lewy Bodies [63] and Corticobasal Degeneration [64], and the TMEM106B genotype has been found to modulate the protective effect of cognitive reserve in genetic forms of frontotemporal dementia [65].

It remains to be seen whether connectivity measures are reliable enough to be useful as diagnostic or longitudinal biomarkers, but they are beginning to reveal a complex interaction between brain structure and function in neurodegeneration.

## Big data and artificial intelligence

The field of neuroimaging has led the scientific community in open science initiatives, particularly in the creation of large repositories of open data [66, 67]. The most widely used datasets in the field of neurodegeneration are the Alzheimer’s Disease Neuroimaging Initiative (ADNI) [68], the Dominantly Inherited Alzheimer Network (DIAN) [69], the Parkinson’s Progression Markers Initiative (PPMI) [70], the Genetic Frontotemporal Initiative (GenFI) [8], and the ARTFL-LEFTDS Longitudinal Frontotemporal Lobar Degeneration (ALLFTD) cohort [71]. Given some of these datasets consist of over 1000 participants, they have attracted the attention of groups working with Artificial Intelligence (AI) methods.

AI describes a set of mathematical tools for identifying relationships and patterns within data and is particularly suitable for the complex and non-linear relationships found in neuroimaging data [72]. These methods need large datasets to pick up subtle patterns and to work out what might be ‘signal’ and what might be ‘noise’. Machine learning methods are a subset of AI tools that have been used to predict the path of cognition in people with early signs of cognitive change with reasonable success [73, 74], and in one study these findings were replicable in a second cohort of patients [75]. Hence, machine learning methods are showing some promise in predicting cognitive change but do need to be translated to the clinical setting, which may require a cultural shift by clinicians who will need to adapt to using new information available from AI algorithms [76].

More complex models have been applied to the challenge of diagnosis using a subset of machine learning methods called ‘deep learning’ algorithms. Theoretically, these methods can detect a wider variety of features within a dataset, and they do indeed achieve a high accuracy (up to 96.0%) in the diagnosis of people with dementia [11]. But this improved accuracy comes at a cost of interpretability, ie it is not clear what parts of the scan are being used to assign people to a diagnostic group. It could be argued that the lack of transparency doesn’t matter, as long as the answer is correct. But, if these methods are going to be transferred into routine clinical practice, the clinician must have an answer as to ‘why’ the algorithm assigns someone to having dementia or not. It may be there are other explanations for the brain changes picked out by the algorithm that could lead to misclassification, for example a person may have hippocampal sclerosis causing hippocampal atrophy and not Alzheimer’s disease. A few emerging methods may address the challenge of interpretation in deep learning, such as the DeepLight method that has successfully been applied to functional MRI data in the human connectome project [77]. Another way to overcome these challenges is by brute force—larger sets of training datasets in the tens or hundreds of thousands of scans, rather than the few hundred to a couple of thousand that we currently have available. These larger datasets will allow the deep learning algorithm to have ‘seen’ a particular abnormality multiple times before, even if it is uncommon, and the error rates will fall as a result. Even so, it may be that doctors are reluctant to trust an algorithm they do not fully understand.

## Translation

The advances in imaging shedding light on diagnosis, prognosis and pathology are welcome in the research world, but as yet they have made very little impact on clinical practice.

One critical issue in achieving this is the standardisation of methodologies for neuroimaging data collection, preprocessing and analysis. Standard work schemes are beginning to emerge driven by the Organisation for Human Brain Mapping [78] and large datasets such as the UK biobank (https://imaging.ukbiobank.ac.uk/) and the Human Connectome Project [79]. However, even with standard methods there remain significant regulatory hurdles that are required to properly assess and register these methods as medical products. This process takes time and energy, probably beyond the scope of the scientists who develop them for their own academic work. Companies would usually take on and scale up such products but may be reluctant to take on methods that are already in the public domain through (hugely valuable) open science initiatives. It may, therefore, fall to clinicians to take the lead to ensure that methods considered standard in academic circles are translated to clinical practice.

## The next big thing…?

It is, of course, notoriously difficult to predict future trends in any field. But one modality of MRI that is receiving increasing attention is Quantitative Susceptibility Mapping (QSM). This technique is sensitive to iron, calcium and other magnetic substances [80]. The deposition of iron measured using QSM has been linked with cognition in Alzheimer’s disease [81] and Parkinson’s disease [82]. In Alzheimer’s disease, iron is found in the plaques and tangles that characterise the disease, though there is still some work required to establish what exactly is being identified, for example whether ferrous or ferric iron is picked up by QSM. Although iron chelation therapy is now being trialled in some of these condition, the drugs can cause life-threatening complications of agranulocytosis and neutropenia which tempers ones enthusiasm for them [83], but nevertheless this imaging modality may yet shed light on the role of iron in the aetiology of neurodegeneration, and prove to be a useful non-invasive biomarker.

## Conclusions

In conclusion, the field of neuroimaging is maturing from the simple measurement of volume and structure, to a host of methods that can identify better methods for monitoring disease progression and pathology as well as help us to better understand patterns of neurodegeneration, and uncover mechanisms that protect cognitive function in the face of neuropathology. As these methods mature, it will be up to clinicians to lead their translation to the clinical world. If that can be achieved, the revolution in neuroimaging will be complete.
